# Involvement of community health workers in antimicrobial stewardship interventions and programmes: a scoping review

**DOI:** 10.1136/bmjgh-2025-020257

**Published:** 2025-10-27

**Authors:** Enrique Castro-Sánchez, Aina Huguet-Torres, Aina María Yáñez-Juan, Miquel Bennasar-Veny

**Affiliations:** 1 NIHR Health Protection Research Unit in Healthcare Associated Infection and Antimicrobial Resistance, Imperial College London, London, UK; 2 Research Group on Global Health, University of the Balearic Islands, Palma, Spain; 3 Department of Nursing and Physiotherapy, University of the Balearic Islands, Palma, Spain

**Keywords:** Delivery of Health Care, Global Health, Health Personnel, Universal Health Care

## Abstract

**Introduction:**

Antimicrobial resistance (AMR) poses a global health threat, especially in low-income and middle-income countries. Community health workers (CHWs) are key actors in infection management and health promotion; however, their involvement in antimicrobial stewardship (AMS) remains unclear. This study aims to assess CHWs’ roles in AMS, examine their training and support, and evaluate outcomes regarding antibiotic use and resistance.

**Methods:**

A scoping review was conducted to explore the roles, education, training and antimicrobial-related outcomes of CHWs. The review followed the Preferred Reporting Items for Systematic Reviews and Meta-Analyses extension for Scoping Reviews guidelines, with the protocol registered in Open Science Framework. Databases including PubMed, EMBASE and CINAHL were searched for studies published in English and Spanish. A narrative synthesis was applied to the identified articles, with the Integrated Quality Criteria for the Review of Multiple Study Designs and Quality Assessment Tool for Studies with Diverse Designs tools employed to assess the risk of bias.

**Results:**

Eight studies were identified, conducted in Uganda, Tanzania, Zambia, Pakistan, Bangladesh and Kenya. CHWs were involved in various AMS activities, such as infection prevention, detection and treatment of conditions such as pneumonia and gastrointestinal infections. CHWs contributed to health education, antibiotic prescription (when authorised) and improved adherence to guidelines. Positive outcomes included reduced inappropriate antibiotic use and increased community awareness of AMR. However, the effectiveness of these interventions varied depending on the local context and resources.

**Conclusions:**

CHWs play an important role in promoting responsible antibiotic use and addressing AMR, particularly in underserved settings. Strengthening their training, expanding selected roles and responsibilities and improving support mechanisms should be considered to enhance their potential contribution to AMS programmes. Further research is needed to optimise CHW interventions and evaluate their long-term impact.

**WHAT IS ALREADY KNOWN ON THIS TOPIC**

- Community health workers (CHWs) are vital in health systems worldwide to deliver last-mile preventive and curative services, including managing infections. Their involvement in antimicrobial stewardship (AMS) remains unexplored.

**WHAT THIS STUDY ADDS**

- Our study addresses a significant gap in the literature by systematically reviewing and synthesising the global evidence on how CHWs are involved in AMS efforts, and what policy and system-level interventions may be needed to support such roles. CHWs contribute to AMS activities, including infection prevention, detection and treatment. Their interventions, such as health education and antibiotic prescription, reduce inappropriate antibiotic use and increase community awareness of drug-resistant infections.

**HOW THIS STUDY MIGHT AFFECT RESEARCH, PRACTICE OR POLICY**

- To harness the potential of CHWs, policymakers and healthcare providers should expand their roles in antibiotic stewardship programmes, invest in training and support, and conduct further research on their long-term impact.

## Introduction

 Antimicrobial resistance (AMR) or infections which are difficult to treat pose a severe global threat to human and animal health.^1^ The WHO has identified AMR as one of the top 10 public health threats facing humanity.^2^ Recent data indicate that AMR is directly or indirectly associated with approximately 1.2 million and 4.9 million deaths, respectively.^3^ However, these infections are unequally and unjustly distributed worldwide, with the burden of AMR disproportionately affecting health services, patients and citizens in low-income and middle-income countries (LMICs) despite access to antibiotics in those countries affected by issues of supply and quality, among others.^4 5^

Antibiotics are often used as short-term solutions to mitigate structural deficits in LMICs, particularly in contexts where socioeconomic determinants or health and limited access to preventive services drive high infection rates.^6^ Moreover, gaps in healthcare workers’ training and availability often contribute to the overuse and misuse of antimicrobials.^7^ To address the threat of AMR, coordinated and comprehensive interventions under the umbrella of ‘antimicrobial stewardship’ (AMS) have been implemented globally.^8^ AMS is defined by the WHO as ‘a coherent set of actions which promote the responsible use of antimicrobials’.^8^ This definition applies to actions at the individual, national and global levels, and across human health, animal health and the environment. AMS interventions often include multiple components, such as education for healthcare workers, patients and citizens; surveillance and feedback; support from leaders; accountability; and synergies with infection prevention and control (IPC) providers.^9^

In many healthcare systems, especially in LMICs, the challenge of AMR is compounded by staffing shortages, with estimates that by 2030, 18 million healthcare workers will be needed.^10^ To address such shortages, novel professional cadres have been developed and deployed to meet clinical and social care needs as well as public health goals.^11^ Many countries have introduced community health workers (CHWs), or healthcare providers who live in the community they serve and receive a lower level of formal education and training than other professional healthcare workers, such as nurses and doctors.^12^

CHWs serve and care for vulnerable populations and those placed in positions of vulnerability by socioeconomic, cultural or political determinants, tailoring their contributions to local contexts.^13^ CHW programmes have thus adopted several regional and national-specific shapes, driven by various requirements, with examples such as the Brazilian Programa Saúde da Família,^14^ Ethiopia’s health extension workers,^15^ the Behvarzs of Iran,^16^ female healthcare workers in Pakistan,^17^ Bangladeshi community and village health workers and Shashthya Shebikas,^18^ Auxiliary Nurse-Midwives in India,^19^ and finally the South African array of CHWs.^20^

CHWs typically manage a wide range of preventive and therapeutic interventions and clinical conditions,^21^ including Ebola^22^ or COVID-19 infection,^23^ with the existing evidence suggesting that the clinical outcomes achieved by CHWs are excellent,^24^ once adequate support and supervision,^25^ education and continuous development, and skill-, staff- and patient-mix^26^ are in place. Health problems managed by CHWs often include infections or situations where antibiotics are warranted, such as the identification and treatment of respiratory tract infections among infants and children.^27^ Given the increasing emphasis on AMS and the pivotal role that CHWs play in delivering primary care, particularly in LMICs, a comprehensive review of their involvement in AMS is both timely and necessary.^8^

Such review can inform future policies, training strategies and health system strengthening efforts by identifying existing practices, gaps and opportunities for integration. Current national and international AMS competencies for healthcare workers centre on those formally educated in medicine and surgery, nursing and midwifery and pharmacy.^28^,^30^ Additionally, there is a lack of research on the knowledge, attitudes and practices of CHWs regarding AMS.^31^ Furthermore, while the existing literature emphasises a multiprofessional approach to AMS involving the entire continuum of healthcare workers,^32^ there is limited guidance on how to effectively integrate CHWs into these efforts.

This scoping review aims to synthesise the existing evidence on the role of CHWs in AMS initiatives globally, with a focus on the practices and outcomes of CHWs in AMS interventions worldwide. Specifically, it aims to answer the following questions:

- What are the practices of CHWs in antimicrobial stewardship?
- Which educational and training approaches have been used to support high-quality CHW engagement in AMS interventions?
- What are the AMS outcomes for CHWs, other professionals, patients, citizens and health systems?

## Methods

### Eligibility criteria

#### Study design

We conducted a scoping review including all study types (quantitative, qualitative and mixed methods) without design restrictions. Studies were included if they reported on the roles, education, and training and antimicrobial-related outcomes of CHWs at multiple levels. This review followed the Preferred Reporting Items for Systematic Reviews and Meta-Analyses extension for Scoping Reviews (PRISMA-ScR) guidelines.^33^ The study protocol was registered in the Open Science Framework (OSF) on 11 October 2023 (https://doi.org/10.17605/OSF.IO/5C6MS).

#### Search strategy

We used a search string related to CHWs, as outlined in a reference document by the WHO.^21^ We used broad terms to perform the search to incorporate all studies with relevant information on the topic. The keywords, search terms and search strategy used are listed in online supplemental appendix 1.

We systematically searched the following databases: PubMed/MEDLINE, EMBASE, CINAHL, Cochrane and Campbell Collaboration to extract the manuscripts. The search strategy was constructed to include descriptors and keywords relevant to the population (CHWs) and activities of interest (AMS and antimicrobial management). Although no language restriction was applied during the search, we only included studies in English and Spanish, as these were the languages spoken by the research team. The same search terms were used in all the databases whenever possible. The last search on these databases was performed on 17 September 2024.

We reviewed the grey literature using Grey Matters (https://greymatters.cda-amc.ca) and checked the reference lists of articles examined during the review to identify potentially relevant additional references. The last search on this database was performed on 4 August 2025.

#### Study screening, selection and reporting

Titles, abstracts and full texts were screened independently by two reviewers (EC-S and AH-T), with a third author (MB-V) resolving discrepancies. One reviewer (AH-T) extracted content from all full texts, with a cross-check of a random 25% sample by a second reviewer (EC-S), and a third author (MB-V) arbitrated any discrepancies. Covidence software (https://www.covidence.org) was used at all stages of the review. A standardised electronic data extraction form was used to collect key information from each study. Extracted variables included citation details, study setting, objectives, design and population served. Specific attention was given to the roles and scope of CHWs in AMS, including whether AMS was part of their core responsibilities or an added activity. We categorised CHW actions into prevention (eg, health education, vaccination), detection (eg, diagnosis, surveillance) and response (eg, treatment, referral, follow-up).

Additional variables included CHW training (duration, content, trainers), support mechanisms (eg, supervision, checklists, mobile apps), measurement approaches (process, outcome or economic), main results, stakeholder views and funding sources. A full description of the extracted variables is provided in online supplemental appendix 2.

### Participants

The review adopted the broad definition of CHWs used by the WHO^21^ and accompanying evidence map (see search string in online supplemental appendix 1). Although there is a wide range of CHWs worldwide, they all share the following characteristics: (1) focusing on culturally appropriate healthcare provision; (2) living in the community they serve with a general understanding of these communities’ language and culture; (3) being paid a salary and (4) having a level of formal education and training which is lower or shorter than other professional healthcare workers such as nurses, physicians or pharmacists.

### Antimicrobial stewardship

The term ‘AMS’ refers to an organisational or healthcare system-wide strategy for promoting and regulating the appropriate use of antibiotics to maintain their long-term efficacy.^8^ We considered the components of AMS routinely advocated by national or international public health agencies, national action plans on AMR or scientific societies. These components include education and training for healthcare workers and communities; surveillance and reporting of antibiotic use and resistance; strategies to support optimised prescribing practices and IPC measures. A full list and description of AMS components considered in this review is provided in online supplemental appendix 3.

We broadly categorised the involvement of CHWs in AMS components to evaluate their potential impact and integration into AMS efforts. This categorisation, developed by consensus of the researchers, would allow us to understand how CHWs contribute to or support AMS objectives in their communities and healthcare settings. In terms of antimicrobial outcomes, we aimed to assess indicators such as infection rates, antibiotic prescription practices, patient health outcomes and antibiotic resistance trends.

### Primary and secondary outcomes

The primary outcome was an understanding of the characteristics, roles and activities of CHWs in relation to AMS interventions and programmes. Secondary outcomes included the identification of education and support received by CHWs to demonstrate competencies in AMS, and a description of metrics related to patients (eg, health status, patient satisfaction, quality of life), own CHWs (eg, job satisfaction related to AMS) and health system (eg, costs, resource utilisation).

### Patient and public involvement

There was no patient and/or public involvement in the design and conduct of this scoping review.

### Strategy for data synthesis

We constructed a narrative synthesis of the findings of this review. An iterative process was used to identify the relationships emerging across the studies reviewed. An evidence map and matrix were developed to summarise the data.

### Risk of bias (quality) assessment

The methodological quality of included studies was initially assessed using the Integrated Quality Criteria for the Review of Multiple Study Designs (ICROMS).^34^ This tool employs a structured scoring system comprising 33 items grouped into seven categories, including both design-specific and general methodological criteria. A decision matrix is used to determine whether individual studies meet minimum methodological standards. For studies with designs not compatible with ICROMS, we applied the Quality Assessment Tool for Studies with Diverse Designs (QATSDD).^35^ This tool includes 14 core criteria for quantitative studies, each scored on a 4-point scale ranging from 0 (not at all) to 3 (fully met), for a maximum possible score of 42.

Two reviewers (EC-S and AH-T) independently completed the relevant quality assessment for each selected study. A third reviewer (MB-V) moderated disagreements. Given the exploratory nature of this scoping review, no studies were excluded based on their quality scores. Our objective was to capture the breadth of available evidence and to describe the diversity of CHW involvement in AMS interventions, irrespective of methodological rigour. The results of the quality assessments were used to contextualise the findings and are further discussed in the limitations section.

## Results

### Search results

**Figure 1:**
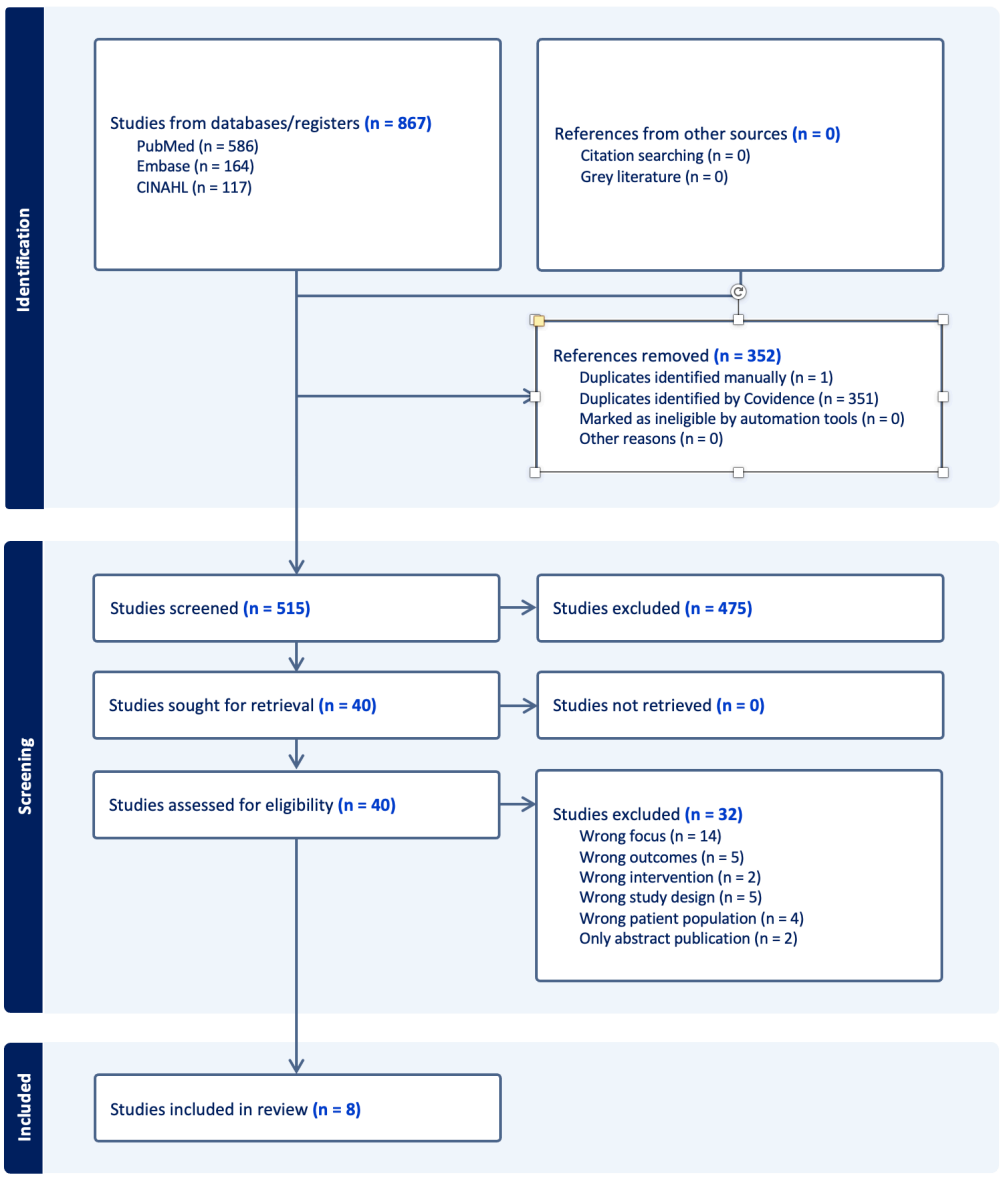
Article screening and selection using the Preferred Reporting Items for Systematic Reviews and Meta-­ Analyses extension for Scoping Reviews flow diagram.

The PRISMA-ScR flow diagram (figure 1) presents the results of this study. Initially, 867 records were identified through electronic database searches. Following deduplication and title, abstract and full-text screening, eight studies were included in the final synthesis.

### Characteristics of included studies

The relevant information for each study included in the review is presented in table 1. This information is summarised below according to key categories. The eight studies identified that examined the involvement of CHWs in antibiotic use employed diverse research designs, including qualitative,^36^,^38^ quantitative^39^,^41^ and mixed-methods approaches.^36 42^ The studies were conducted in various countries, including Tanzania,^38^ Zambia,^42^ Bangladesh,^37^ Uganda,^36 41 43^ Kenya^39^ and Pakistan.^40^

**Table 1:** Characteristics of studies included in the review (n=8)

Author, year, N, country	Design	Aim	Population(s) served	Clinical area	Roles and responsibilities	Training	Support received	Results/ performance and impact
Musoke *et al,*^36^ 2020 N=227 Uganda	Mixed methods	Strengthen AMS/AMR awareness via a One Health approach	Children (under 5 years)	Malaria, diarrhoea and pneumonia	Preventive, detection	Workshop sessions on AMR/AMS/IPC introduction, WHO AMR competency framework, prudent antibiotic use, Uganda Clinical Guidelines (UCG) and Microguide app, One Health, hand hygiene and knowledge sharing using COM-B model.	CHW who supervises or mentors clinical practice on AMS; Community of Practice.	204 CHWs (90%) reported improved practices after training, including increased handwashing (81%) and encouraging proper medication adherence (67%). Qualitative evaluation confirmed improved AMR prevention practices, particularly in animal husbandry, such as observing antibiotic withdrawal periods and reducing human-prescribed antimicrobials.
Nahar *et al.*, ^37^ 2020 N=46 Bangladesh	Qualitative	Explore understanding of antibiotic use, AMR awareness and patient demand perception.	Adults	Sepsis	Preventive, detection, dispensing	Semiqualified providers: 1 year of health-related training (formal providers) Unqualified providers: without any training (informal providers).	–	Antibiotics are seen as powerful medicines effective against most diseases, including viruses, with expensive ones considered strongest. While some providers were aware of AMR, many confused it with side effects, and responsibility for misuse was often shifted to patients and animal owners.
Rakhshani *et al*,^40^ 2022 N=177, 10 CHW Pakistan	Quantitative	Assess and describe antibiotic dispensing/prescribing practices and factors among community-based healthcare workers to inform community AMS programmes.	All population	Upper respiratory infection (URI), gastrointestinal, urinary tract infection, skin infection, pelvic inflammatory disease.	Preventive, detection, dispensing	–	–	CHWs reported the highest rates of antibiotic requests, with 40% of patients seeking antibiotics, and were more likely to regularly use Ceftriaxone. Over 50% of healthcare workers, including physicians, nurses and midwives, were familiar with antibiotic prescribing guidelines, and 80% of CHWs had heard of AMR.
Nyamu *et al*,^39^ 2021 N=3014 Kenya	Quantitative	Evaluate adherence to Integrated Management of Childhood Illnesses guidelines in prescriptions by CHW.	All population	URI	Preventive, detection	Interactive training on AMS, understanding AMR, factors influencing upper respiratory tract infection (URTI) visits, reasons for high antibiotic use. Evidence-based URTI management per Kenyan guidelines.	Other professionals who supervise or mentor; Clinical practice guidelines or checklists adapted for use; Education.	Antimicrobial prescriptions reduced by 44% in children under 5 years within 2 weeks post-intervention, and by 18% at weeks 8–9. In children over 5 years, prescriptions decreased by 18% and 8%, respectively.
Musoke *et al.*,^43^ 2021 N=8 Uganda	Qualitative	Access, use and disposal of antimicrobials among humans and animals	Children (under 5 years)	Malaria, diarrhoea and pneumonia	Dispensing	–	–	Access to antimicrobials from public health facilities was reported by CHWs and farmers, but challenges (ie, long travel distances) were common. Some CHWs disposed of expired antimicrobials at these facilities, mainly those involved in childhood illness treatment.
Graham *et al.,*^42^ 2016 N=90 Zambia	Mixed methods	Assess rational use of antibiotics by CHWs and factors influencing adherence to administration by caregivers.	Children	Suspected pneumonia	Detection, dispensing	6 days of training in integrated Community Case Management.	Clinical practice guidelines or checklists adapted for use by CHWs.	CHWs adhered to treatment guidelines for 92% of children, but only 65% of antibiotics were prescribed for children with confirmed pneumonia. 46% of caregivers fully adhered to the 5-day amoxicillin course, increasing to 76% when considering those who gave treatment for 3–5 days.
Virhia *et al,*^38^ 2023 N=44 Tanzania	Qualitative	Understand factors affecting translation AMR knowledge into effective action among HCWs.	All population	Sepsis	Preventive	Short health training course.	–	There was variation in training and understanding of AMR. Structural challenges (lack of resources, infrastructure) common, with patients often blamed for improper antibiotic use, reflecting a gap in understanding underlying AMR drivers. Findings highlight the urgent need for targeted training to improve healthcare responses and community care.
Ciccone *et al,*^41^ 2024 N=65 Uganda	Quantitative	To determine the impact of C reactive protein (CRP) measurement on antibiotic use among children presenting with febrile acute respiratory illness to CHW	Children	URI	Detection, dispensing	5-day intensive training prior to the study	Provision of tools, government and external programme backing, and dedicated funding that enabled the intervention	A CRP-guided algorithm led to a 24.6% reduction in antibiotic use without increasing adverse outcomes. High adherence and feasibility among CHWs were reported, with strong alignment to clinical guidance.

AMR, antimicrobial resistance; AMS, antimicrobial stewardship; CHW, community health worker; HCWs, healthcare workers; IPC, infection prevention and control.

### Quality/risk of bias of the selected studies

Four studies employed designs that were compatible with the ICROMS tool. Among these, two studies^37 43^ fully met all mandatory ICROMS criteria, while the other two^38 39^ only partially met them. In particular, the reflexivity criterion (Q6) was not addressed in Virhia *et al.*^38^ The remaining four studies^3640^,^42^ employed designs not supported by the ICROMS and were therefore assessed using the QATSDD tool. Although there were methodological limitations, such as limited justification of design choices, lack of ethical reporting or minimal stakeholder involvement, most studies achieved acceptable scores (69%–86%). A summary of the quality appraisal, including the tool applied, score obtained, compliance with ICROMS mandatory criteria (where applicable), and study-specific observations, appears on table 2.

**Table 2:** Methodological quality assessment and risk of bias across included studies

Study	Design	Appraisal tool	Score (% of max)	Observations
Graham *et al* 2016^42^	Cross-sectional mixed methods	QATSDD	33/42 (79%)	Well-structured analysis but minimal integration of qualitative/quantitative data.
Musoke *et al* 2020^36^	Post-only descriptive evaluation	QATSDD	34/42 (81%)	Strong contextual description and adequate analytical justification.
Nahar *et al* 2020^37^	Qualitative	ICROMS	31/36 (86%)	Fully met all ICROMS mandatory criteria (6/6); strong in reflexivity, sampling justification and data analysis transparency.
Nyamu *et al* 2021^39^	Non-controlled before–after	ICROMS	25/36 (69%)	Partially met mandatory ICROMS criteria (3/5); lacked design justification and ethical reporting.
Musoke *et al* 2021^43^	Qualitative	ICROMS	30/36 (83%)	Met 5/6 ICROMS mandatory criteria; high methodological clarity, partial reporting of reflexivity.
Rakhshani *et al* 2022^40^	Cross-sectional	QATSDD	33/42 (79%)	Clear aim, context and sampling; limited stakeholder involvement.
Virhia *et al* 2023^38^	Qualitative	ICROMS	30/36 (83%)	Met 5/6 ICROMS mandatory criteria; did not address reflexivity (Q6).
Ciccone *et al* 2024^41^	Stepped-wedge cluster randomised trial	QATSDD	36/42 (86%)	High-quality pragmatic trial.

ICROMS, Integrated Quality Criteria for the Review of Multiple Study Designs; QATSDD, Quality Assessment Tool for Studies with Diverse Designs.

### Clinical area or condition requiring antibiotic use by CHWs

The studies indicate that CHWs play a relevant role in managing a range of clinical conditions that often require antibiotic use, including URI, pneumonia, gastrointestinal infections, diarrhoea and other diseases such as malaria. For example, CHWs are actively involved in treating pneumonia in children under 5,^41 42^ as well as in managing gastrointestinal infections and pneumonia.^36 43^ Other CHWs are involved in caring for patients across all age groups, treating multiple diseases, such as upper respiratory tract infections, gastrointestinal infections and urinary tract infections,^40^ while other CHWs focus specifically on diseases, such as upper respiratory tract infections.^39^ CHWs also play a key role in treating severe diseases, such as malaria,^36 43^ and for managing sepsis in the population.^37 38^

### CHW roles and responsibilities

CHWs played multifaceted roles in promoting the appropriate use of antibiotics. These roles encompassed preventive measures, where CHWs engaged in activities aimed at preventing infections and promoting the appropriate use of antibiotics. These activities include health promotion, education on antibiotic use and resistance, vaccination campaigns and ensuring access to clean water and sanitation facilities.^36^,^40^

CHWs were also involved in the detection and response to infections. In this domain, CHWs may be responsible for tasks such as assessing symptoms, classifying diseases and providing treatment and education for a broad range of clinical episodes, including respiratory infections,^37 39 40^ sepsis, diarrhoea and pneumonia,^42 43^ as reflected in detail in the previous section, and overall facilitating timely and appropriate antibiotic treatments. They also use relatively complex tools such as C reactive protein point-of-care measurements.^41^ Following detection, CHWs contribute to the response by providing treatment, dispensing or prescribing antibiotics when authorised, interpreting diagnostic tests and assisting other healthcare professionals.^37 40 42 43^

Furthermore, the studies suggest that the use of antibiotics is often considered a core competency for CHWs, highlighting the importance of their role in the AMS. However, the extent of CHWs’ involvement in antibiotic-related activities varies, with studies reporting that CHWs have taken on new or expanded roles in this area, such as managing suspected pneumonia in children under 5 years old.^42^

### Training and support

The literature highlights the importance of providing CHWs with adequate training and support to enable them to effectively contribute to AMS activities. CHW engagement in AMS varies considerably depending on country context, programme maturity and training. The nature and duration of training programmes described also varied across studies; some reported brief training programmes, while others described more comprehensive interventions. For example, one study involved a 6-day training programme on integrated Community Case Management, covering clinical algorithms, diagnosis and treatment guidelines.^42^ Another study used workshops covering a wide range of topics, including AMR awareness, IPC and the use of clinical guidelines.^36^

### CHW performance and impact on antibiotic-related areas

The performance and impact of CHWs in antibiotic-related areas were not generally reported in the studies analysed (missing in four of them). When included, a range of evaluation indicators was available, with a dominance of outcome-focused metrics (three instances, the most frequently used metric), including adherence to treatment guidelines or antibiotic prescription accuracy, reflecting a focus on compliance and quality of care.

Several studies reported positive AMR-related outcomes associated with CHW interventions, including improved adherence to treatment guidelines, thereby reducing the risk of treatment failure and the development of antibiotic resistance,^42^ reduced inappropriate prescription and dispensing of antibiotics,^39 41^ and education of citizens about the importance of responsible antibiotic use and the consequences of antibiotic resistance, offering overall increased community awareness of AMR challenges.^36^ The same study also showed improvements in CHW practices, including increased handwashing, better medication adherence and advancements in AMR prevention, particularly in animal husbandry.^36^

The improvements varied across the different contexts researched, with significant and more modest reductions in inappropriate antibiotic use observed across studies. These variations may be explained by factors such as the specific design of the CHW intervention, the population served, the prevalent clinical conditions and the wider health system context. For example, some studies showed greater awareness of antibiotic resistance among CHWs,^40^ while others reported challenges in adherence to the guidelines, as evidenced by antibiotics being prescribed for children with pneumonia.^42^

Overall, these findings suggest the significant role already played by CHWs in optimising antibiotic use and their potential contribution to AMS programmes if this domain of their practice were to be formalised and enhanced. However, gaps in understanding AMR persist, such as confusion between antibiotic resistance and side effects.^37 38^

## Discussion

To our knowledge, this is the first review to outline the participation of CHWs in AMS programmes and interventions, their education and knowledge, and the outcomes achieved. Our review identified eight studies from Uganda, Tanzania, Zambia, Pakistan, Bangladesh and Kenya, illustrating the relevance of CHW involvement in AMS. The findings highlight the diverse roles of CHWs in antibiotic use, including prevention, detection and treatment of common conditions that require antibiotic use, such as pneumonia, gastrointestinal infections and sepsis. Their functions are often adapted to the needs of the populations they serve, ranging from health promotion and community education to direct patient care, including symptom assessment and disease management.

Given that CHWs are often and frequently the first point of contact with the formal health and care system in LMICs,^44^ their engagement in AMS can significantly impact antibiotic use and AMR at the community level. Despite their proximity to vulnerable populations and their role in healthcare, their specific contributions to AMS remain unexplored.

About roles and responsibilities of CHWs in antibiotic use, they contribute to AMS through various strategies, including health promotion, community education, vaccination campaigns and infection prevention measures. These efforts have been recognised as effective in reducing the incidence of infection and, consequently, the need for antibiotics. However, several implementation barriers hinder the full potential of CHW-led interventions, including inconsistent training, lack of standardised guidelines and limited resources.^41^ Inadequate supervision and shortages of essential medical supplies further constrain CHWs’ ability to implement AMS strategies.

In addition to prevention, CHWs play a key role in detecting and responding to infections. In many LMICs, they are trained to assess symptoms, classify diseases and administer treatment when authorised. These efforts are recognised as effective strategies for reducing infections and minimising inappropriate antibiotic use, particularly in LMICs.^44^ However, there is notable variability in their competencies and practical implementation of CHW-led interventions, often dictated by differences in national policies, available resources and the level of integration into formal healthcare systems. This preventive approach aligns with international recommendations to strengthen primary healthcare in vulnerable communities.^45^ Addressing these discrepancies would require structured training programmes and well-defined scopes of practice to ensure that CHWs can contribute effectively to AMS.^46^

Many studies in this review reported the positive impact of CHW interventions on AMS, including improved adherence to treatment guidelines, reduced inappropriate antibiotic use and enhanced community awareness of AMR. However, the magnitude of these effects varied significantly across different settings. For instance, in areas where CHWs received strong institutional support, such as access to supervision, decision-making frameworks and ongoing education, there was a clear reduction in the misuse of antibiotics.^39 43^ Conversely, in regions with weak health infrastructure, CHWs face difficulties adhering to AMS guidelines due to limited diagnostic tools, lack of clear prescribing regulations and cultural perceptions favouring antibiotic use. Future research should further explore the contextual factors that influence the effectiveness of CHW-led AMS interventions, particularly in settings where regulatory and logistical constraints limit the ability to enforce responsible antibiotic use.^47 48^

The magnitude of these effects varied substantially across different contexts and programmatic designs. Ensuring that CHWs are equipped with high-quality training would be critical for their role in AMS. Current evidence suggests that while short-term workshops can address immediate knowledge gaps, more comprehensive competency-based training models are needed for sustainable impact. Training strategies that integrate simulation-based learning, peer mentoring and interprofessional collaboration have been particularly effective in strengthening CHWs’ skills. For example, approaches incorporating interactive case discussions and scenario-based learning have demonstrated better retention of AMS principles than traditional lecture-based methods. Additionally, culturally adapted training programmes that acknowledge local beliefs and practices regarding antibiotic use can enhance CHWs’ ability to communicate effectively with the community.

A pressing gap in current training is the lack of standardised AMS curricula tailored for CHWs. While national and international AMS competency frameworks exist for physicians, nurses and pharmacists, CHWs have not been systematically included in these frameworks. Given their expanding role in healthcare delivery, incorporating CHWs into WHO-recommended AMS education would be essential. This variability also underscores a need for further research to identify pedagogical approaches that best support CHWs in delivering AMS interventions effectively.

Finally, ensuring that CHWs receive adequate training and continuous support would be critical to their effective contribution to AMS efforts. While initial training equips CHWs with foundational knowledge, ongoing supervision, mentorship and access to decision-support tools significantly improve their adherence to antibiotic guidelines. However, for many CHWs, refresher training and structured supervision are either unavailable or inconsistently implemented.^49^ One key factor in strengthening CHW training is the use of competency-based educational models. Approaches that integrate practical simulations, interactive case discussions and peer learning have demonstrated higher knowledge retention and better application of AMS principles in clinical settings,^50^ although data for CHWs are lacking. However, despite the effectiveness of these methods, their implementation remains inconsistent across LMICs.^51^ Recent WHO guidance reinforces these findings, recommending the integration of AMR into CHW pre-service and refresher training through structured content, practical methodologies and supervision.^52^

### Impact

The scoping review provides evidence of the positive impact of CHW interventions on antibiotic use. CHWs can contribute to improved adherence to treatment guidelines, reduced inappropriate antibiotic use and increased community awareness of antibiotic resistance. These findings highlight the potential of CHWs to contribute significantly to optimising antibiotic use and combating the global challenge of AMR. Further research is needed to explore the factors influencing the effectiveness of CHW interventions and strategies to maximise their impact on AMS.

### Limitations

The scoping review has some limitations. Patients or members of the public were not involved in the design, conduct, reporting or dissemination plans of this scoping review. The scarce number, geographical concentration, varied quality and methodological rigour of the studies should be considered when interpreting our results. The lack of papers from much of the globe, including Europe, the Americas and Southeast Asia may reflect publication or researcher biases, as well as local legislative and normative frameworks that prevent CHWs from engaging with antibiotics. The findings may not be extrapolated to regions with CHWs but not represented in our review. This review may also be subject to language bias, as only studies published in English and Spanish were included. Finally, the included studies varied in methodological quality, which may affect the reliability of some findings. Caution is advised, especially where conclusions are based on evidence with higher risk of bias.

### Implications for research and public health practice

We propose the following implications aligned with the objectives of this review:

1. Strengthen and systematise CHW practices in AMS: Policymakers and health programme implementers should consider supporting the integration of CHWs into AMS activities, particularly in underserved settings. Their involvement in prevention, detection and response tasks has shown promise, but consistent guidance, role definition and appropriate remuneration remain critical to enhance their contributions.
2. Improve training approaches and ongoing support mechanisms: Ensuring that CHWs receive high-quality, context-sensitive training is essential to their effective engagement in AMS interventions. Future programmes should invest in tailored pedagogical methods, mentorship structures and supportive tools that enable CHWs to apply AMS principles confidently and accurately.
3. Generate further evidence on AMS-related outcomes and implementation pathways: Additional research is needed to evaluate the long-term impact of CHW-led AMS interventions, both on antibiotic use and on broader public health outcomes. Particular attention should be paid to understanding implementation mechanisms, system-level facilitators and barriers, and strategies for effective scale-up and adaptation to diverse contexts.

## Conclusions

This scoping review underscores the potential of CHWs to contribute to the optimal use of antibiotics. By leveraging their unique position within communities, CHWs can effectively promote appropriate antibiotic practices, improve patient outcomes and contribute to the fight against AMR. Further research is needed to explore the long-term impact of CHW interventions and identify best practices for integrating CHWs into antibiotic stewardship programmes.

## Supplementary material

10.1136/bmjgh-2025-020257 online supplemental appendix 1

10.1136/bmjgh-2025-020257 online supplemental appendix 2

10.1136/bmjgh-2025-020257 online supplemental appendix 3

## Data Availability

All data relevant to the study are included in the article or uploaded as supplementary information.

## References

[1] Murray CJL (2022). BSAC Vanguard Series: Tracking the global rise of antimicrobial resistance. J Antimicrob Chemother.

[2] World Health Organization (2021). Antimicrobial resistance. https://www.who.int/news-room/fact-sheets/detail/antimicrobial-resistance.

[3] Antimicrobial Resistance Collaborators (2022). Global burden of bacterial antimicrobial resistance in 2019: a systematic analysis. Lancet.

[4] Iskandar K, Molinier L, Hallit S (2021). Surveillance of antimicrobial resistance in low- and middle-income countries: a scattered picture. *Antimicrob Resist Infect Control*.

[5] Wasan H, Reeta KH, Gupta YK (2024). Strategies to improve antibiotic access and a way forward for lower middle-income countries. J Antimicrob Chemother.

[6] Chandler CIR (2019). Current accounts of antimicrobial resistance: stabilisation, individualisation and antibiotics as infrastructure. Palgrave Commun.

[7] Holmes AH, Moore LSP, Sundsfjord A (2016). Understanding the mechanisms and drivers of antimicrobial resistance. Lancet.

[8] World Health Organization (2019). Antimicrobial Stewardship Programmes in Health-Care Facilities in Low- and Middle-Income Countries. A Practical Toolkit.

[9] Tadesse BT, Ashley EA, Ongarello S (2017). Antimicrobial resistance in Africa: a systematic review. BMC Infect Dis.

[10] World Health Organization (2016). Working for health and growth: investing in the health workforce. report of the high-level commission on health employment and economic growth. https://www.who.int/publications/i/item/9789241511306.

[11] Dovlo D (2004). Using mid-level cadres as substitutes for internationally mobile health professionals in Africa. Hum Resour Health.

[12] Glenton C, Javadi D, Perry HB (2021). Community health workers at the dawn of a new era: 5. Roles and tasks. Health Res Policy Syst.

[13] APHA (2019). Support for community health workers to increase health access and to reduce health inequities. https://www.apha.org/policies-and-advocacy/public-health-policy-statements/policy-database/2014/07/09/14/19/support-for-community-health-workers-to-increase-health-access-and-to-reduce-health-inequities.

[14] Ministerio de Saúde (2024). Estratégia saúde da família. https://www.gov.br/saude/pt-br/composicao/saps/estrategia-saude-da-familia.

[15] Haileamlak A, Ataro I (2023). The Ethiopian Health Extension Program (HEP) is Still Relevant After 15 Years of Implementation Although Major Transformation is Essential to Sustain Its Gains and Relevance. Ethiop J Health Sci.

[16] Shams L, Zamani Fard M, Nasiri T (2023). Community health workers (Behvarz) in primary health care: a qualitative inductive content analysis of challenges. Aust J Prim Health.

[17] Hafeez A, Mohamud BK, Shiekh MR (2011). Lady health workers programme in Pakistan: Challenges, achievements and the way forward. J Pak Med Assoc.

[18] Afsana K, Hussein J, McCaw-Binns A, Webber R (2012). Maternal and Perinatal Health in Developing Countries.

[19] Pyone T, Karvande S, Gopalakrishnan S (2019). Factors governing the performance of Auxiliary Nurse Midwives in India: A study in Pune district. PLoS ONE.

[20] Rwafa-Ponela T, Eyles J, Christofides N (2020). Implementing without guidelines, learning at the coalface: a case study of health promoters in an era of community health workers in South Africa. Health Res Policy Syst.

[21] World Health Organization (2010). What do we know about community health workers? a systematic review of existing reviews (human resources for health observer series no. 19). https://iris.who.int/bitstream/handle/10665/340717/9789241512022-eng.pdf?sequence=1.

[22] Perry HB, Dhillon RS, Liu A (2016). Community health worker programmes after the 2013-2016 Ebola outbreak. Bull World Health Organ.

[23] Nawaz S, Moon KJ, Vazquez R (2023). Evaluation of the Community Health Worker Model for COVID-19 Response and Recovery. J Community Health.

[24] Rawal LB, Kharel C, Yadav UN (2020). Community health workers for non-communicable disease prevention and control in Nepal: a qualitative study. BMJ Open.

[25] Crigler L, Gergen J, Perry H, Perry H, Crigler L (2014). Developing and strengthening community health worker programs at scale: A reference guide and case studies for program managers and policy makers (chap. 10).

[26] Dubois C-A, Singh D (2009). From staff-mix to skill-mix and beyond: towards a systemic approach to health workforce management. Hum Resour Health.

[27] Méllo LMB de D e, Santos RC dos, Albuquerque PC de (2023). Community Health Workers: what do international studies tell us?. Ciênc saúde coletiva.

[28] Courtenay M, Hawker C, Gallagher R (2025). The application of antimicrobial stewardship knowledge to nursing practice: A national survey of United Kingdom pre-registration nursing students. J Adv Nurs.

[29] Wu JH-C, Khalid F, Langford BJ (2021). Community pharmacist prescribing of antimicrobials: A systematic review from an antimicrobial stewardship perspective. Can Pharm J (Ott).

[30] World Health Organization (2018). WHO Competency Framework for Health Workers’ Education and Training on Antimicrobial Resistance.

[31] Mudenda S, Hankombo M, Saleem Z (2020). Knowledge, attitude, and practices of community pharmacists on antibiotic resistance and antimicrobial stewardship in lusaka, zambia. Public and Global Health.

[32] Pereira NR, Castro-Sanchez E, Nathwani D (2017). How can Multi-Professional Education Support Better Stewardship?. Infect Dis Rep.

[33] Tricco AC, Lillie E, Zarin W (2018). PRISMA Extension for Scoping Reviews (PRISMA-ScR): Checklist and Explanation. Ann Intern Med.

[34] Zingg W, Castro-Sanchez E, Secci FV (2016). Innovative tools for quality assessment: integrated quality criteria for review of multiple study designs (ICROMS). Public Health (Fairfax).

[35] Harrison R, Jones B, Gardner P (2021). Quality assessment with diverse studies (QuADS): an appraisal tool for methodological and reporting quality in systematic reviews of mixed- or multi-method studies. BMC Health Serv Res.

[36] Musoke D, Kitutu FE, Mugisha L (2020). A One Health Approach to Strengthening Antimicrobial Stewardship in Wakiso District, Uganda. Antibiotics (Basel).

[37] Nahar P, Unicomb L, Lucas PJ (2020). What contributes to inappropriate antibiotic dispensing among qualified and unqualified healthcare providers in Bangladesh? A qualitative study. BMC Health Serv Res.

[38] Virhia J, Gilmour M, Russell C (2023). “If You Do Not Take the Medicine and Complete the Dose…It Could Cause You More Trouble”: Bringing Awareness, Local Knowledge and Experience into Antimicrobial Stewardship in Tanzania. Antibiotics (Basel).

[39] Nyamu N, Mbatia F, Van den Hombergh P (2021). Burden of upper respiratory tract infections in primary care facilities and excessive antimicrobial over-prescription: A community-oriented primary care project in rural Kenya. Afr J Prim Health Care Fam Med.

[40] Rakhshani NS, Kaljee LM, Khan MI (2022). A Formative Assessment of Antibiotic Dispensing/Prescribing Practices and Knowledge and Perceptions of Antimicrobial Resistance (AMR) among Healthcare Workers in Lahore Pakistan. Antibiotics (Basel).

[41] Ciccone EJ, Hu D, Preisser JS (2024). Point-of-care C-reactive protein measurement by community health workers safely reduces antimicrobial use among children with respiratory illness in rural Uganda: A stepped wedge cluster randomized trial. PLoS Med.

[42] Graham K, Sinyangwe C, Nicholas S (2016). Rational use of antibiotics by community health workers and caregivers for children with suspected pneumonia in Zambia: a cross-sectional mixed methods study. BMC Public Health.

[43] Musoke D, Namata C, Lubega GB (2021). Access, use and disposal of antimicrobials among humans and animals in Wakiso district, Uganda: a qualitative study. J of Pharm Policy and Pract.

[44] Rowe AK, De Savigny D, Lanata CF (2005). How can we achieve and maintain high-quality performance of health workers in low-resource settings?. Lancet.

[45] Schriger SH, Knowles M, Daglieri T (2024). Barriers and Facilitators to Implementing an Evidence-Based Community Health Worker Model. *JAMA Health Forum*.

[46] Alhassan JAK, Wills O (2024). Public health surveillance through community health workers: a scoping review of evidence from 25 low-income and middle-income countries. BMJ Open.

[47] World Health Organization (2019). Declaration of astana: global conference on primary health care: Astana, Kazakhstan, 25 and 26 october 2018. https://iris.who.int/handle/10665/328123.

[48] Scott K, Beckham SW, Gross M (2018). What do we know about community-based health worker programs? A systematic review of existing reviews on community health workers. Hum Resour Health.

[49] Olaniran A, Smith H, Unkels R (2017). Improving the competence of community health workers in low-and middle-income countries: a systematic review of training interventions. Hum Resour Health.

[50] Davey P, Marwick CA, Scott CL (2017). Interventions to improve antibiotic prescribing practices for hospital inpatients. Cochrane Database Syst Rev.

[51] Mathew P, Ranjalkar J, Chandy SJ (2020). Challenges in Implementing Antimicrobial Stewardship Programmes at Secondary Level Hospitals in India: An Exploratory Study. Front Public Health.

[52] World Health Organization (2025). Inclusion of Antimicrobial Resistance in Training Programmes for Community Health Workers: Technical Brief.

