# Supplementary material for: Involvement of community health workers in antimicrobial stewardship interventions and programmes: a scoping review
Source: BMJ Glob Health. 2025 Oct 27;10(10):e020257. doi: 10.1136/bmjgh-2025-020257 (PMC12557733; doi:10.1136/bmjgh-2025-020257)
Supplement: online supplemental appendix 1 [file bmjgh-10-10-s001.docx]

**APPENDIX 1**

(“antimicrobial resistance” OR “antimicrobial stewardship”) AND

(“health auxiliary”[tw] OR “frontline health workers”[tw] OR “frontline health worker”[tw] OR “midwife”[tw] OR “Midwifery”[tiab] OR “midwives”[tw] OR “Birth Attendant”[tw] OR “Midwives”[tw] OR “outreach worker”[tw] OR “outreach workers”[tw] OR “lay health worker”[tw] OR “lay health workers”[tw] OR “promotora”[tw] OR “promotoras”[tw] OR “village health worker” OR “village health workers”[tw] OR “volunteer health worker”[tw] OR “volunteer health workers”[tw] OR “voluntary health workers”[tw] OR “voluntary health worker”[tw] OR “community health agent”[tw] OR “community health agents”[tw] OR “health promoter”[tw] OR “health promoters”[tw] OR “Community Health Workers”[Mesh] OR “community health worker”[tw] OR “community health workers”[tw] OR “community health aide”[tw] OR “community health aides”[tw] OR “community health nursing”[tw] OR “community health nurses”[tw] OR “community health nurse”[tw] OR “community health officers”[tw] OR “community health officer”[tw] OR “community health volunteer”[tw] OR “community health volunteers”[tw] OR “community health distributors”[tw] OR “community health distributor”[tw] OR “community health surveyors”[tw] OR “community health surveyor”[tw] OR “community health assistants”[tw] OR “community health assistant”[tw] OR “community health promoters”[tw] OR “community health promoters”[tw] OR “community IMCI”[tw] OR “community volunteer”[tw] OR “community volunteers”[tw] OR “health extension workers”[tw] OR “health extension worker”[tw] OR “village health volunteer”[tw] OR “village health volunteers”[tw] OR “Community Health Nursing”[Mesh] OR “close-to-community provider”[tw] OR “close-to-community providers”[tw] OR “community-based practitioner”[tw] OR “community-based practitioners”[tw] OR “lady Health worker”[tw] OR “lady Health workers”[tw] OR “barefoot doctor”[tw] OR “Community Practitioners”[tw] OR “Community Practitioner”[tw] OR “community-based practitioners”[tw] OR “community-based practitioner”[tw] OR “promotoras de salud”[tw] OR “agentes de saúde”[tw] OR “rural health auxiliaries”[tw] OR “traditional birth attendants”[tw] OR “traditional birth attendant”[tw] OR “Activista”[tw] OR “Agente comunitario de salud”[tw] OR “Agente comunitário de saúde”[tw] OR “Anganwadi”[tw] OR “Animatrice”[tw] OR “Barangay health worker”[tw] OR “Barangay health workers”[tw] OR “Basic health worker”[tw] OR “Basic health workers”[tw] OR “Brigadista”[tw] OR “Colaborador voluntario”[tw] OR “Community drug distributor”[tw] OR “Community drug distributors”[tw] OR “Community health agent”[tw] OR “Community health agents”[tw] OR “Community health promoter”[tw] OR “Community health promoters”[tw] OR “Community health representative”[tw] OR “Community health representatives”[tw] OR “Community health volunteer”[tw] OR “Community health volunteers”[tw] OR “Community resource person”[tw] OR “Female multipurpose health worker”[tw] OR “Female multipurpose health worker”[tw] OR “Health promoter”[tw] OR “Health promoters”[tw] OR “Kader”[tw] OR “Monitora”[tw] OR “Mother coordinator”[tw] OR “Outreach educator”[tw] OR “Outreach educators”[tw] OR “Promotora”[tw] OR “Shastho shebika”[tw] OR “Shastho karmis”[tw] OR “Sevika”[tw] OR “Village health helper”[tw] OR “Village drug-kit manager”[tw] OR “Accompagnateur”[tw] OR “Accredited Social Health Activist”[tw] OR “Animator”[tw] OR “ASHA”[tw] OR “Auxiliary Nurse”[tw] OR “Auxiliary Nurse-midwife”[tw] OR “Bridge-to-Health Team”[tw] OR “Behvarz”[tw] OR “Care Group”[tw] OR “Care Groups”[tw] OR “Care Group Volunteer”[tw] OR “Care Group Volunteers”[tw] OR “Community Case Management Worker”[tw] OR “Community Case Management Workers”[tw] OR “Community Health Agent”[tw] OR “Community Health Agents”[tw] OR “Community Health Care Provider”[tw] OR “Community Health Care Providers”[tw] OR “Community HealthCare Provider”[tw] OR “Community HealthCare Providers”[tw] OR “Community Health Extension Worker”[tw] OR “Community Health Extension Workers”[tw] OR “Community Health Officer”[tw] OR “Community Health Officers”[tw] OR “Community Surveillance Volunteer”[tw] OR “Community Surveillance Volunteers”[tw] OR “Family Health Worker”[tw] OR “Family Health Workers”[tw] OR “Family Planning Agent”[tw] OR “Family Planning Agents”[tw] OR “Family Welfare Assistant”[tw] OR “Family Welfare Assistants”[tw] OR “Female Community Health Volunteer”[tw] OR “Female Community Health Volunteers”[tw] OR “Health Agent”[tw] OR “Health Agents”[tw] OR “Health Assistant”[tw] OR “Health Assistants”[tw] OR “Health Extension Worker”[tw] OR “Health Extension Workers”[tw] OR “Health Surveillance Assistant”[tw] OR “Health Surveillance Assistants”[tw] OR “Kader”[tw] OR “Lead Mother”[tw] OR “Malaria Agent”[tw] OR “Malaria Agents”[tw] OR “Maternal and Child Health Worker”[tw] OR “Maternal and Child Health Workers”[tw] OR “Mobile Clinic Team”[tw] OR “Mobile Clinic Teams”[tw] OR “Nutrition Agent”[tw] OR “Nutrition Agents”[tw] OR “Nutrition Counselor”[tw] OR “Nutrition Counselors”[tw] OR “Peer Educator”[tw] OR “Peer Educators”[tw] OR “Shasthya Shebika”[tw] OR “Socorrista”[tw])
