# Supplementary material for: Involvement of community health workers in antimicrobial stewardship interventions and programmes: a scoping review
Source: BMJ Glob Health. 2025 Oct 27;10(10):e020257. doi: 10.1136/bmjgh-2025-020257 (PMC12557733; doi:10.1136/bmjgh-2025-020257)
Supplement: online supplemental appendix 2 [file bmjgh-10-10-s002.docx]

**Appendix 2. Standardised data extraction form**

1. Source

1.1 Citation, year of publication

2. Methods

2.1 Country of study

2.2 Aim of study

2.3 Study design

3. Participants

3.1 Numbers

3.2 Terminology to Describe Community Health Worker Role

3.3 Setting of practice (community- or facility-based)

4. Population

4.1 Population cared for or served by community health workers (i.e., adults, children, both, rural community, farmers, etc)

5. Area of community health worker involvement in AMS

5.1 Infection or activity (malaria, tuberculosis, etc.)

5.2 Is AMS a core or extra activity?

5.3 Scope of practice and role components

5.3.1 Prevent (i.e., health promotion, education, vaccination, etc, adequate nutrition, access to clean water, effective sanitation systems, triage, etc.)

5.3.2 Detect (i.e., diagnosis, physical examination, history taking, disease surveillance, etc)

5.3.3 Respond (i.e., dispense, prescribe, treat, management ordering tests, interpret tests, follow up (surgery or postpartum sepsis), assist others, etc.)

6. Training provided for community health workers to engage in AMS interventions

6.1 Duration, trainers, content, place

7. Support provided for community health workers to engage in AMS interventions

7.1 Supervisors, other professionals, checklists, guidelines, apps, etc.

8. Measures

8.1 Measurements tools for process, outcome, and economic

9. Results

9.1 Key conclusions

10. Views of participants or other stakeholders

11. Funding source

11.1 Governmental or non-governmental
