# Supplementary material for: Involvement of community health workers in antimicrobial stewardship interventions and programmes: a scoping review
Source: BMJ Glob Health. 2025 Oct 27;10(10):e020257. doi: 10.1136/bmjgh-2025-020257 (PMC12557733; doi:10.1136/bmjgh-2025-020257)
Supplement: online supplemental appendix 3 [file bmjgh-10-10-s003.docx]

**Appendix 3. Antimicrobial stewardship components considered in the review**

- **Education and training**: Educational programs to inform healthcare workers, patients, and the community about appropriate use of antimicrobials, emphasising reducing misuse and overuse.
- **Surveillance and reporting**: Systems for tracking antibiotic usage and reporting data on antimicrobial resistance.
- **Optimised prescribing practices**: Strategies to ensure the appropriate prescription of antibiotics, including guidance on selecting the correct antibiotic, dosing, and duration of treatment based on clinical guidelines.
- **Infection Prevention and Control (IPC)**: Collaboration with IPC programs to reduce infection rates, including hygiene practices, vaccination efforts, and other measures to prevent infections.
